# CTX-M–Type β-Lactamases Affect Community *Escherichia coli* Treatment, Greece

**DOI:** 10.3201/eid1006.031031

**Published:** 2004-06

**Authors:** Spyros Pournaras, Alexandros Ikonomidis, Danai Sofianou, Athanassios Tsakris, Antonios N. Maniatis

**Affiliations:** * University of Thessalia, Larissa, Greece;; † Hippokration University Hospital, Thessaloniki, Greece;; ‡ University of Athens, Athens, Greece

**Keywords:** CTX-M–Type β-Lactamases, E. coli, Escherichia coli treatment, Greece

**To the Editor:** In recent years, a new group of extended-spectrum β-lactamases (ESBLs), the CTX-M–type enzymes, has emerged among *Enterobacteriaceae* (*1*). These enzymes are much more active against cefotaxime than against ceftazidime and exhibit greater susceptibilty to tazobactam than to clavulanate. Currently, the CTX-M family includes at least 30 alleles, which may be clustered on the basis of sequence similarity into four major evolutionary lineages (*2*). These enzymes were first detected in South America, Germany, and France, and subsequent reports found them to be in several European countries as well as in the Far East and North and South America (*1**–**5*). All of these studies have described the production of CTX-M–type β-lactamases in *Enterobacteriaceae* that have been isolated from patients with hospital infections, whereas surveys assessing countrywide prevalence of CTX-M clusters in community-acquired infections have not been carried out. We report the dissemination of various CTX-M-type enzymes in clinical isolates of *Escherichia coli* recovered from community-acquired infections in two large regions of Greece.

The microbiology databases of two healthcare systems in Greece that serve as tertiary care centers for their region (University Hospital of Larissa and Hippokration University Hospital of Thessaloniki) were prospectively searched from January to September 2003. From almost 75,000 outpatient visits, we tested *E. coli* isolates that were recovered from patients with community-acquired infections and classified as ESBL producers by the E-test ESBL screen method with cefotaxime and ceftazidime plus clavulanate. Community-acquired *E. coli* infections were defined as those contracted outside a hospital environment for persons with no history of hospitalization, surgery, or outpatient care during the previous 30 days.

ESBL-positive isolates for which the cefotaxime MICs were at least eightfold higher than those of ceftazidime by agar dilution were saved and stored at –70°C. Results of antimicrobial susceptibility tests and ESBL screening and confirmatory tests were used to characterize the phenotypes of the isolates. Molecular analysis included polymerase chain reaction (PCR) detection with primers producing an 873-bp amplicon of the *bla*_CTX-M_ gene (*6*), sequencing on both strands of PCR products, pulsed-field gel electrophoresis of *Xba*I chromosomal digests, plasmid analysis, and transferability experiments of cefotaxime resistance.

Treatment outcomes were assessed by reviewing the medical records of all patients for whom a culture yielded a strain of *E. coli* producing a CTX-M–type ESBL. Patients’ data included demographic details, presence of existing illness, symptoms, laboratory test results, history of surgery, and exposure to extended-spectrum cephalosporins <30 days before the positive culture. The antimicrobial treatment regimen was recorded, including the agent or agents administered, the duration of treatment, and clinical response.

During the study period, 14 community-acquired *E. coli* isolates (10 in the region of Larissa and 4 in the region of Thessaloniki) were recovered; the E-test ESBL screen test confirmed that these isolates were ESBL producers, and cefotaxime MICs were at least eightfold higher than those of ceftazidime. A CTX-M–type determinant was detected by PCR in 10 isolates (6 from Larissa and 4 from Thessaloniki). Sequencing their amplicons revealed that three of them were CTX-M-1 producers, and seven were CTX-M-3 producers. Genotyping showed that all of these isolates were unrelated. MICs of cefotaxime were always >128 µg/mL, whereas MICs of ceftazidime ranged from 0.5 µg/mL to 16 µg/mL. All isolates were sensitive to cefoxitin and piperacillin/tazobactam. However, the two laboratories reported them as being ESBL producers and recommended that β-lactam antibiotics, with the exception of carbapenems, not be used in their treatment. Several isolates exhibited additional resistance to co-trimoxazole, ciprofloxacin, tetracycline, or gentamicin, but all were susceptible to the other aminoglycosides. The *bla*_CTX-M_ determinants were transferable to *E. coli* by conjugation in all but one case, along with other antimicrobial resistance determinants, with transfer frequencies that ranged from 8.3 x 10^–5^ to 2.2 x 10^–2^. Transconjugants contained one to three plasmids of varying size, ranging from 10 to 130 kb.

CTX-M–positive isolates were recovered from five children and five adults. Patients did not have typical risk factors, except for a chronic hematologic malignancy in one patient. Nine of the patients had severe urinary tract infections and received courses of amikacin or ciprofloxacin. One female patient had a purulent perianal infection. Results of blood and wound cultures from samples obtained at admission yielded the CTX-M–positive *E. coli* strain. She was given amikacin and clindamycin, and her condition gradually improved.

β-lactam antibiotics are the most common antimicrobial agents used in the community setting. The documented CTX-M–positive isolates exhibited plasmid-mediated resistance that affected the antimicrobial activity of all penicillins and cephalosporins as well as of several alternative antimicrobial agents used to treat community-acquired *E. coli* infections. The spread of CTX-M–positive bacteria considerably changes the way we think about treating community-acquired infections and limits the oral antibiotics that may be administered. This finding has major implications for treating children, who should not be given fluoroquinolones and tetracyclines.

The observation that different *bla*_CTX-M_ alleles, located on plasmids of different sizes, were involved in clinical infections caused by distinct *E. coli* clones implies that CTX-M enzymes may become widespread in the community. A possible association of *bla*_CTX-M_ genes with insertion sequences like IS*Ecp1B* might have contributed to the enhanced expression and mobilization of *bla*_CTX-M_ genes among *E. coli* isolates (*7*). The apparent dissemination of CTX-M producers could represent a substantial barrier in the treatment of community-acquired infections. Additionally, severely ill patients treated in the outpatient setting may transmit such resistant organisms to hospitalized patients.

## References

[1] Tzouvelekis LS, Tzelepi E, Tassios PT, Legakis NJ. CTX-M-type β-lactamases: an emerging group of extended-spectrum enzymes. Int J Antimicrob Agents. 2000;14:137–42. 10.1016/S0924-8579(99)00165-X 10720804

[2] Navarro F, Miró E. Update of CTX-M-type β-lactamases. Rev Med Microbiol. 2002;13:63–73. 10.1097/00013542-200204000-00003

[3] Edelstein M, Pimkin M, Palagin I, Edelstein I, Stratchounski L. Prevalence and molecular epidemiology of CTX-M extended-spectrum β-lactamase-producing *Escherichia coli* and *Klebsiella pneumoniae* in Russian hospitals. Antimicrob Agents Chemother. 2003;47:3724–32. 10.1128/AAC.47.12.3724-3732.2003 14638473 PMC296190

[4] Mushtaq S, Woodford N, Potz N, Livermore DM. Detection of CTX-M-15 extended-spectrum β-lactamase in the United Kingdom. J Antimicrob Chemother. 2003;52:528–9. 10.1093/jac/dkg353 12888591

[5] Smith Moland E, Black JA, Hossain A, Hanson ND, Thomson KS, Pottumarthy S. Discovery of CTX-M-like extended-spectrum β-lactamases in *Escherichia coli* isolates from five U.S. states. Antimicrob Agents Chemother. 2003;47:2382–3. 10.1128/AAC.47.7.2382-2383.2003 12821506 PMC161829

[6] Gniadkowski M, Schneider I, Palucha A, Jungwirth R, Mikiewicz B, Bauernfeind A. Cefotaxime-resistant *Enterobacteriaceae* isolates from a hospital in Warsaw, Poland: identification of a new CTX-M-3 cefotaxime-hydrolyzing β-lactamase that is closely related to the CTX-M-1/MEN-1 enzyme. Antimicrob Agents Chemother. 1998;42:827–32. 9559791 10.1128/aac.42.4.827 PMC105550

[7] Poirel L, Decousser JW, Nordmann P. Insertion sequence *ISEcp1B* is involved in expression and mobilization of a *bla*_CTX-M_ β-lactamase gene. Antimicrob Agents Chemother. 2003;47:2938–45. 10.1128/AAC.47.9.2938-2945.2003 12936998 PMC182628

